# Comparison of textbook outcomes and postoperative pain trajectories between reduced-port and conventional robotic distal gastrectomy: a cumulative sum (CUSUM)-adjusted propensity score-matched analysis

**DOI:** 10.1007/s11701-026-03607-y

**Published:** 2026-06-16

**Authors:** Dongwon Lim, Jongmin Han, Juyeong Noh, Si-Hak Lee, Sun-Hwi Hwang, Hanpyo Hong, Jae Hun Chung

**Affiliations:** 1 Department of Surgery, Pusan National University Yangsan Hospital, 20 Geumo-ro, Mulgeum-eup, Yangsan-si, 50612 Kyungsangnam-do Republic of Korea; 2 School of Medicine, Pusan National University, Yangsan, Republic of Korea; 3 Research Institute for Convergence of Biomedical Science and Technology, Pusan National University Yangsan Hospital, Yangsan, Republic of Korea

**Keywords:** Gastric cancer, Robotic gastrectomy, Reduced-port surgery, Textbook outcome, Propensity score matching

## Abstract

To compare textbook outcome (TO) and postoperative pain trajectories between reduced-port robotic distal gastrectomy (rpRDG) and conventional robotic distal gastrectomy (cRDG) for gastric cancer (GC). This retrospective cohort study analyzed patients who underwent robotic distal gastrectomy for GC between September 2022 and March 2026 at a single tertiary center. Cumulative sum (CUSUM) analysis was applied to exclude 30 cases representing the initial learning curve, yielding 107 patients (cRDG, *n* = 60; rpRDG, *n* = 47). Following 1:1 propensity score matching (PSM), 39 matched pairs were analyzed. The primary endpoint was TO achievement; secondary endpoints included operative time, estimated blood loss (EBL), retrieved lymph nodes, and postoperative pain assessed by numeric rating scale (NRS) from postoperative day (POD) 1 to 3. The two groups were well-balanced after PSM (all absolute standardized mean differences < 0.1). TO achievement rates were comparable between groups (cRDG: 79.49% vs. rpRDG: 87.18%, *p* = 0.3621), with no significant differences in operative time (264.97 vs. 246.23 min, *p* = 0.0808), EBL (47.05 vs. 43.21mL, *p* = 0.7088), or retrieved lymph nodes (34.67 vs. 38.92, *p* = 0.2686). A linear mixed-effects model revealed a significant group-by-time interaction in pain scores (*p* = 0.0044), and the rpRDG group reported significantly lower NRS scores on POD 1 (2.62 vs. 3.13, *p* = 0.0155). rpRDG demonstrated comparable surgical and oncologic outcomes to cRDG, with statistically significant reduction in early postoperative pain on POD 1, representing a meaningful marginal gain within an ERAS-optimized setting. These findings support rpRDG as a feasible, patient-centered alternative warranting further multicenter validation.

**Supplementary Information:**

The online version contains supplementary material available at 10.1007/s11701-026-03607-y.

## Introduction

Despite improvements in screening and multimodal treatment, gastric cancer (GC) persists as a leading cause of cancer-related mortality worldwide, with the greater disease burden concentrated in East Asia [1]. Although endoscopy-based screening has increased early detection and reduced mortality while multidisciplinary treatments have expanded, surgery is the principal curative modality for GC [2]. Oncologic outcomes depend on curative gastrectomy with appropriate lymphadenectomy, and contemporary surgical advancements emphasize minimizing invasiveness without compromising oncologic principles [3].

Among minimally invasive approaches, robotic surgery represents the most recent technological evolution. By providing three-dimensional visualization, tremor stabilization, and articulated instrumentation, robotic distal gastrectomy (RDG) allows surgeons to perform precise dissection and suturing. Accumulating clinical evidence has confirmed the safety and feasibility of this approach [4, 5]. However, conventional RDG (cRDG) typically uses a multiport approach. Multiple abdominal incisions can cause postoperative pain, cosmetic issues, and port-related complications, justifying further reductions in abdominal wall trauma [6]. Given that a 2–3 cm umbilical incision is unavoidable for specimen extraction in GC surgery, minimizing additional ports by maximizing this site’s utility supports reduced-port strategies.

Reduced-port and single-port techniques aim to minimize incisions, potentially alleviating postoperative pain, enhancing cosmesis, and decreasing port-related morbidity [4, 6]. In laparoscopic gastrectomy (LG), evidence for reduced-port approaches has grown, including from randomized multicenter trials [7]. For robotic gastrectomy, however, clustering bulky robotic arms in confined spaces poses unique challenges, such as external collisions and intricate docking [4]. Therefore, whether reduced-port RDG (rpRDG) can surmount these constraints to yield outcomes comparable to cRDG—especially in operative efficiency, postoperative morbidity, and oncologic adequacy (resection margins and lymph node yield)—is not clear [8–10]. Although reports on reduced-port techniques exist—including the Korean KLASS-13 study and initial experiences with da Vinci SP and hybrid methods like MILAR—these studies have either compared reduced-port robotic approaches against conventional laparoscopic surgery or reported single-arm feasibility data, without direct comparison against conventional multiport robotic gastrectomy [8–12]. While postoperative pain reduction is frequently cited as a theoretical advantage of reduced-port surgery, objective pain assessments comparing rpRDG and cRDG using validated instruments such as the numeric rating scale have not been reported.

This study, therefore, aimed to assess rpRDG’s clinical feasibility versus cRDG by evaluating short-term surgical outcomes and textbook outcome (TO)—an integrated metric encompassing multiple surgical quality benchmarks—as the primary endpoint, along with postoperative pain as the secondary endpoint, to determine whether rpRDG maintains equivalent perioperative quality while offering potential benefits in postoperative recovery.

## Materials and methods

### Study design and patient selection

This retrospective cohort study included patients who underwent RDG for GC at our institution between September 2022 and March 2026. The study followed the STROCSS 2025 guidelines [13]. Ethical approval was obtained from the Institutional Review Board (IRB) (No. 55-2026-101), with informed consent waived, given the retrospective study design.

**Fig. 1:**
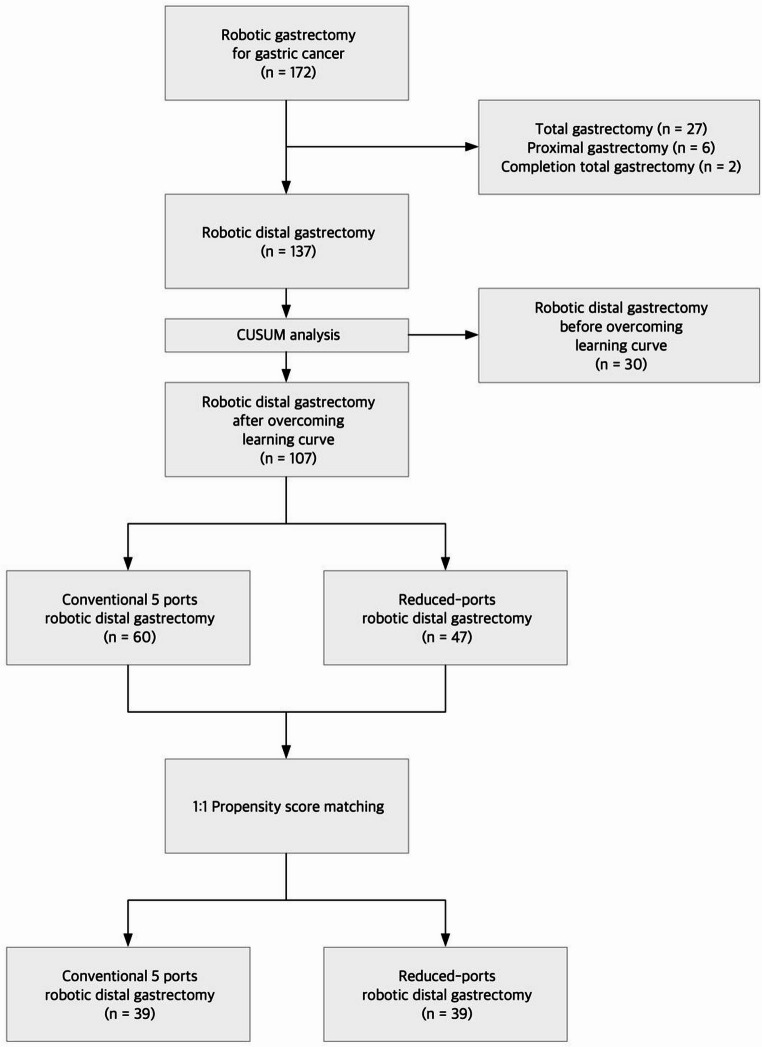
Study flow diagram. The diagram illustrates the patient selection process, exclusion criteria, and propensity score matching, resulting in the final study cohorts for the conventional and reduced-port groups

Among 172 initially screened patients, those with histologically confirmed gastric adenocarcinoma and a preoperative plan for curative-intent robotic gastrectomy were enrolled. The following conditions led to exclusion for procedures other than distal gastrectomy: (1) total gastrectomy (*n* = 27); (2) proximal gastrectomy (*n* = 6); (3) completion total gastrectomy (*n* = 2), leaving 137 eligible patients. To mitigate the learning-curve effect, 30 cases identified via cumulative sum (CUSUM) analysis were excluded (Online resource 1), leaving 107 patients (cRDG: *n* = 60; rpRDG: *n* = 47) for initial analysis. Given that the surgeon had established proficiency in cRDG prior to adopting rpRDG, proficiency transfer was assumed for the rpRDG group; accordingly, only cRDG cases within the learning curve period were excluded. This assumption is supported by the CUSUM trajectory, in which the rpRDG peak value (364.30) was nearly three-fold lower than that of cRDG (1168.67) with earlier stabilization (15th vs. 30th case), consistent with technical adaptation rather than de novo learning. As such, exclusion of the initial rpRDG cases was not considered methodologically warranted. Following 1:1 propensity score matching (PSM), 39 matched pairs were identified for the primary comparative analysis (Fig. 1).

### Surgical procedures and port configuration

Da Vinci robotic systems (Xi, V, or SP, Intuitive Surgical, Sunnyvale, CA, USA) were employed for all RDG procedures. Operations were conducted at a high-volume tertiary referral center performing approximately 300 GC surgeries annually. Surgical indications and oncologic extent followed the Korean Gastric Cancer Association guidelines [14]. Tumors located in the middle or distal stomach were treated with distal gastrectomy, ensuring sufficient proximal margins. The extent of lymphadenectomy was tailored to the preoperative clinical stage determined by endoscopic findings and abdominal CT, with D1 + lymphadenectomy for early GC and D2 lymphadenectomy for advanced disease. One of three reconstruction methods (Billroth I, Billroth II, or Roux-en-Y) was selected intraoperatively at the surgeon’s discretion.

**Fig. 2:**
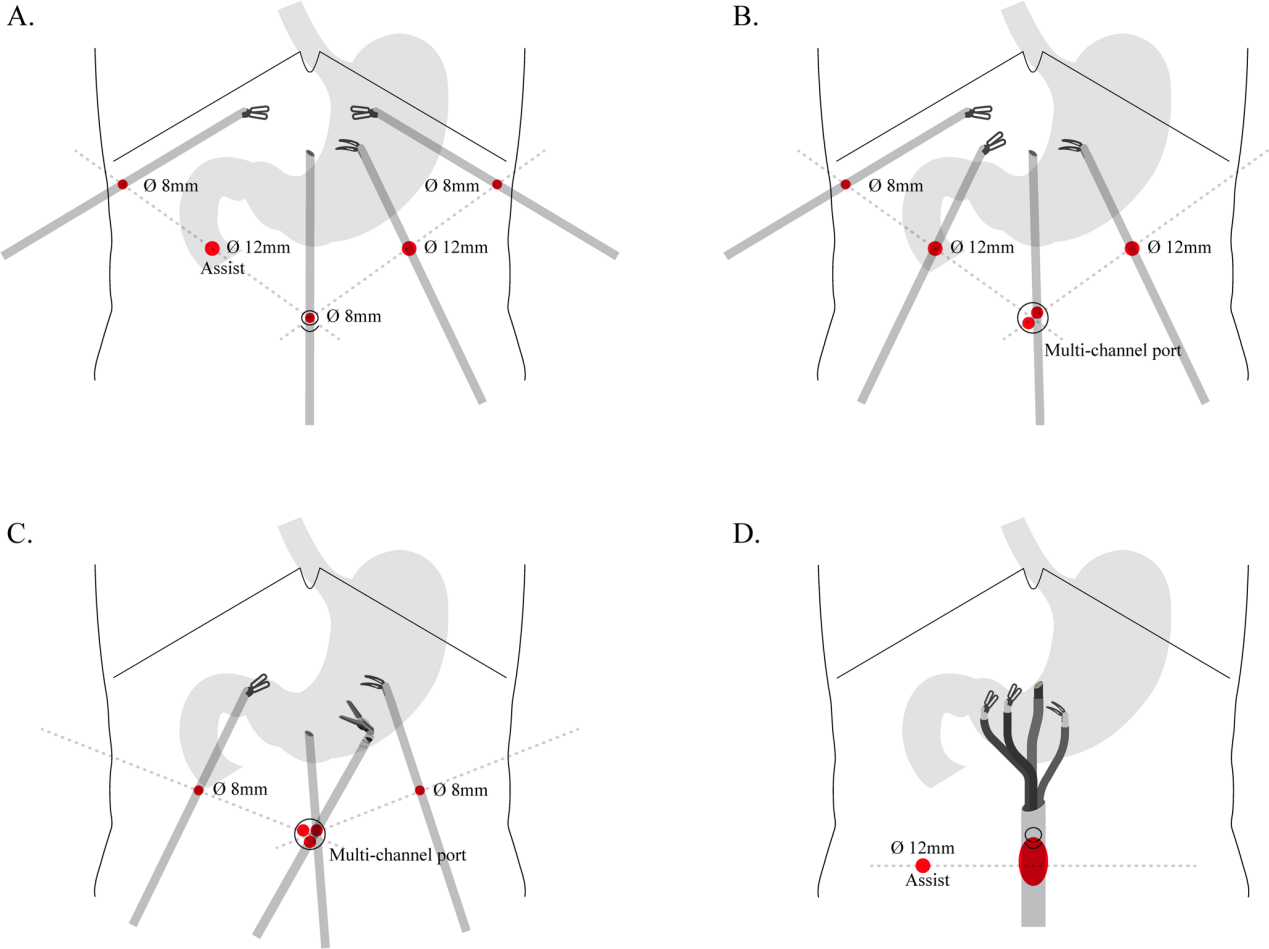
Schematic illustrations of port placement configurations. (**a**) Conventional five-port approach. (**b**) Conventional four-port approach using a multi-channel access device. (**c**) Reduced-port three-port approach using a multi-channel access device, representing the “four-arms-in-three-ports” technique. (**d**) Reduced-port two-port approach using the da Vinci SP system, referred to as the “single-plus-one” method. Red circles indicate the port sites

In the cRDG group, robotic arms were positioned in a standardized V-shaped configuration using anatomical landmarks. Each robotic arm required a dedicated entry site. Arm 1 (8 mm) was placed at the right anterior axillary line, and Arm 4 (8 mm) at the left anterior axillary line. Endoscopic access was established via the umbilical incision, with the choice between two assistant port configurations left to surgeon discretion. In the 5-port setup, a separate 12-mm assistant port was placed at the right mid-clavicular line (Fig. 2a). In the 4-port setup, a multi-channel access device was placed at the umbilical incision (Fig. 2b). In this configuration, the assistant introduced essential materials (e.g., gauze and sutures) through the accessory channel of the umbilical port. Importantly, even in the 4-port setup, only the endoscope occupied the umbilical port for robotic control, thereby maintaining the same wide triangulation as in the 5-port configuration.

In the rpRDG group, strategies were implemented to reduce the number of skin incisions by consolidating robotic arm placement. For procedures using multiport robotic systems (da Vinci Xi or V), a “four-arms-in-three-ports” approach was adopted (Fig. 2c). A multi-channel access device was placed at the umbilical incision to accommodate Arm 2, Arm 3 (8-mm endoscope), and an assistant channel for the delivery of surgical materials. Two 8-mm robotic ports were inserted at the right and left mid-clavicular lines for Arms 1 and 4, respectively, to achieve triangulation. This setup enabled full four-arm functionality with only three skin incisions. For procedures using the single-port robotic system (da Vinci SP), a “single-plus-one” configuration was employed (Fig. 2d). A dedicated single-port cannula was inserted through the umbilical incision, housing the camera and three fully articulating robotic arms. Stapling and clip application necessitated placement of one supplementary 12-mm laparoscopic port, given the current unavailability of robotic staplers and advanced energy devices on the SP system. Assistant port placement was tailored to the reconstruction technique to optimize workflow. For Billroth I reconstruction, the assistant port was positioned in the left upper quadrant to provide a direct linear path for stapler insertion during gastroduodenostomy. For Billroth II or Roux-en-Y reconstruction, it was placed in the right upper quadrant to facilitate jejunal limb mobilization and gastrojejunostomy formation.

### Outcome measures

This study compared clinical outcomes between patients undergoing rpRDG and those undergoing cRDG. TO achievement served as the primary endpoint, encompassing all of the following criteria: (1) negative pathologic resection margins (R0); (2) Lymph node yield of ≥ 16; (3) absence of severe complications (Clavien–Dindo grade ≥ II); (4) no requirement for reoperation or interventional radiology within 30 days; (5) no ICU readmission; (6) hospital length of stay ≤ 21 days; (7) no readmission within 30 days after discharge; (8) no 30-day postoperative mortality [15]. Secondary endpoints included operative time (min), estimated blood loss (mL), number of retrieved lymph nodes, conversion rate to open surgery, and postoperative pain. As part of the enhanced recovery after surgery (ERAS) protocol, all patients in both groups received scheduled intravenous acetaminophen (1000 mg/100 mL every 6 h) from the day of surgery through POD 3 as the baseline analgesic regimen. Numeric rating scale (NRS)-based pain scores were recorded daily by trained nursing staff from POD 1 through POD 3. Rescue analgesics, such as propacetamol hydrochloride or pethidine hydrochloride, were administered only upon the patient’s request if the pain was not adequately controlled by the baseline regimen, and the number of rescue doses was recorded as a supplementary pain indicator [16, 17]. The frequency of rescue analgesic administration was dichotomized as fewer than two doses (< 2) versus two or more doses (≥ 2) per patient during the postoperative course, as repeated administration (≥ 2 doses) was considered to reflect a clinically meaningful requirement for additional analgesia beyond the baseline regimen. These parameters were selected to evaluate procedural efficiency, surgical quality, and early recovery with the reduced-port approach.

### Statistical analysis

SAS software (version 9.4; SAS Institute Inc., Cary, NC, USA) was used for all statistical analyses. Perioperative and short-term surgical outcomes were compared between the cRDG and rpRDG groups. Given the non-normal distribution of most continuous variables, these are reported as medians with interquartile ranges (IQRs); categorical variables are summarized as frequencies and proportions. In the unmatched cohort, between-group comparisons for continuous variables employed the Mann–Whitney U test; categorical variables were examined using the Pearson chi-square test or Fisher’s exact test, as appropriate.

To minimize baseline imbalances and mitigate confounding, propensity score matching (PSM) was applied. Propensity scores were estimated using a logistic regression model including the following baseline covariates: sex, age, American Society of Anesthesiologists (ASA) physical status, body mass index (BMI), and clinical stage of GC. A 1:1 nearest-neighbor algorithm was used to match each rpRDG patients to a cRDG counterpart without replacement based on the logit of the propensity score, with a caliper width equal to 0.1 of the standard deviation of the logit of the propensity score. Covariate balance before and after matching was assessed using standardized mean differences (SMDs), and an absolute SMD < 0.1 was considered indicative of adequate covariate balance. Within the matched cohort, continuous variables were compared using the Wilcoxon signed-rank test, whereas paired categorical variables were analyzed using McNemar’s test or the McNemar–Bowker test, as appropriate. A linear mixed-effects model with group, time, and group×time interaction as fixed effects was fitted to evaluate longitudinal changes in postoperative pain scores. Estimated marginal means were used to conduct post-hoc between-group comparisons at each time point. Statistical significance was defined as a two-sided P-value of less than 0.05.

## Results

### Propensity score matching and baseline patient characteristics

Initially, the total cohort, before any exclusions or matching, consisted of 137 patients: 90 in the conventional RDG (cRDG) group and 47 in the rpRDG group. Baseline characteristics of all 137 patients prior to CUSUM-based exclusion are presented in Table 1 to characterize the overall study population, whereas subsequent comparative analyses were conducted on the post-exclusion cohort of 107 patients (cRDG, *n* = 60; rpRDG, *n* = 47). Table 1 presents the baseline characteristics of the initial cohort, in which several variables were unevenly distributed between the two groups. Comorbidity burden was notably greater in the rpRDG group, with diabetes mellitus (cRDG: 17.78% vs. rpRDG: 34.04%, *p* = 0.0327) and dyslipidemia (cRDG: 21.11% vs. rpRDG: 40.43%, *p* = 0.0165) occurring at significantly higher rates. In contrast, age (*p* = 0.1859), sex (*p* = 0.1921), and BMI (*p* = 0.3791) were comparable between groups.

**Table 1:** Baseline characteristics of patients who underwent robotic distal gastrectomy for gastric cancer —total eligible cohort (before learning-curve exclusion) and propensity score-matched cohort: conventional versus reduced-port approach

variables	Total cohort			After propensity score matching		
	Conventional RDG (*n* = 90)	Reduced-port RDG (*n* = 47)	*p*-value	Conventional RDG (*n* = 39)	Reduced-port RDG (*n* = 39)	*p*-value
Age (years) at OP*	61.50 (56.00-68.75)	65.0 (58.50–70.00)	0.1859	63.00 (57.50–70.00)	65.00 (61.00-70.50)	0.4560
Sex			0.1921			> 0.9999
Male (%)	60 (66.67%)	26 (55.32%)		25 (64.10%)	25 (64.10%)	
Female (%)	30 (33.33%)	21 (44.68%)		14 (35.90%)	14 (35.90%)	
BMI*	23.22 (21.11–25.81)	23.28 (22.33–25.93)	0.3791	23.53 (21.97–25.56)	23.30 (22.77–26.19)	0.5257
Abdominal OP history			0.4169			0.4563
Yes	29 (32.22%)	12 (25.53%)		13 (33.33%)	10 (25.64%)	
No	61 (67.78%)	35 (74.47%)		26 (66.67%)	29 (74.36%)	
ASA score			0.5075			> 0.9999
< 3	85 (94.44%)	43 (91.49%)		38 (97.44%)	38 (97.44%)	
≥ 3	5 (5.56%)	4 (8.51%)		1 (2.56%)	1 (2.56%)	
Comorbidity						
HTN	40 (44.44%)	20 (42.55%)	0.8323	16 (41.03%)	17 (43.59%)	0.8187
DM	16 (17.78%)	16 (34.04%)	0.0327	8 (20.51%)	15 (38.46%)	0.0822
Dyslipidemia	19 (21.11%)	19 (40.43%)	0.0165	9 (23.08%)	16 (41.03%)	0.0894
Cardiovascular	10 (11.11%)	8 (17.02%)	0.3310	5 (12.82%)	6 (15.38%)	0.7449
Cerebrovascular	6 (6.67%)	5 (10.64%)	0.4167	4 (10.26%)	4 (10.26%)	> 0.9999
Nephrology	3 (3.33%)	1 (2.13%)	0.6907	2 (5.13%)	1 (2.56%)	0.5560
Respiratory	7 (7.78%)	3 (6.38%)	0.7657	5 (12.82%)	3 (7.69%)	0.4554

Variables are expressed as a number (%)

Variables with asterisks (*) are expressed as median (Q1-Q3)

RDG = robotic distal gastrectomy; BMI = body mass index; OP = Operation; ASA = American Society of Anesthesiologists; HTN = hypertension; DM = diabetes mellitus

Beyond these clinical imbalances, we identified a potential procedural bias: the majority of cases in the early period of our RDG experience were concentrated in the cRDG group. To address this concentration of early-stage cases in one group and minimize the risk of the comparative results being skewed by the surgeon’s initial learning curve, a CUSUM analysis was performed. The CUSUM curve for operative time identified an inflection point at the 30th case, marking the transition from the learning phase to the proficiency phase (Online Resource 1). Consequently, these initial 30 cases—representing the early, potentially biased phase—were excluded to ensure the analysis focused on outcomes achieved during a stable proficiency period.

Following this exclusion, a 1:1 PSM was conducted to mitigate remaining selection biases and align the covariates between the cRDG group and the proficiency-phase rpRDG cases. This process yielded 39 well-balanced pairs. As presented in Table 1, all baseline characteristics were well balanced after matching, with age showing no statistically significant differences (cRDG: 63.00 years vs. rpRDG: 65.00 years, *p* = 0.4560), sex (*p* > 0.9999), or BMI (*p* = 0.5257). Furthermore, all covariates achieved an absolute SMD of less than 0.1, confirming that the two groups were statistically equivalent for the subsequent analysis of surgical outcomes and pain scores (Online Resource 2).

### Surgical Outcomes

Table 2 summarizes the surgical and oncologic outcomes of the propensity socre-matched cohort. There were no statistically significant differences in operating time between the cRDG and rpRDG groups (264.97 ± 9.17 min vs. 246.23 ± 5.63 min, *p* = 0.0808), suggesting that the reduced-port approach is as efficient as the conventional method in the hands of a proficient surgeon. Similarly, the estimated blood loss showed no significant difference (47.05$\:\pm\:$7.79 mL vs. 43.21$\:\pm\:$5.69 mL, *p* = 0.7088).

**Table 2:** Surgical outcomes after propensity score matching of patients who underwent robotic distal gastrectomy for gastric cancer: conventional versus reduced-port approach

Variables	Conventional RDG (*n* = 39)			Reduced-port RDG (*n* = 39)			*p*-value		
Extent of LND							0.3611		
< D2 (%)	15 (38.46%)			19 (48.72%)					
≥ D2 (%)	24 (61.54%)			20 (51.28%)					
Type of reconstruction							0.7694		
Billroth I (%)	12 (30.77%)			11 (28.21%)					
Billroth II (%)	18 (46.15%)			21 (53.85%)					
Roux-en-Y (%)	9 (23.08%)			7 (17.95%)					
Operation time (min)*	264.97 $\:\pm\:$ 9.17			246.23 $\:\pm\:$ 5.63			0.0808		
Estimated blood loss (mL)*	47.05 $\:\pm\:$ 7.79			43.21 $\:\pm\:$ 5.69			0.7088		
rLN*	34.67 $\:\pm\:$ 2.71			38.92 $\:\pm\:$ 2.77			0.2686		
pT stage							0.1682		
T1	26 (66.67%)				31 (79.49%)				
T2	7 (17.95%)				1 (2.56%)				
T3	4 (10.26%)				5 (12.82%)				
T4	2 (5.13%)				2 (5.13%)				
pN stage							0.4954		
N0	34 (87.18%)				36 (92.31%)				
N1	3 (7.69%)				1 (2.56%)				
N2	1 (2.56%)				0 (0.00%)				
N3	1 (2.56%)				2 (5.13%)				

Variables are expressed as a number (%)

Variables with asterisks (*) are expressed as mean $\:\pm\:$ standard error

RDG = robotic distal gastrectomy; LND = lymph node dissection; rLN = total number of retrieved lymph nodes; pT stage = pathologic T stage; pN stage = pathologic N stage

Regarding oncological radicality, the total number of retrieved lymph nodes was comparable between the groups (34.67$\:\pm\:$2.71 vs. 38.92$\:\pm\:$2.77, *p* = 0.2686), and there were no significant differences in the distribution of pathologic T stage (*p* = 0.1682) or N stage (*p* = 0.4954). The extent of lymph node dissection and types of reconstruction also showed well-balanced distributions between the two groups (*p* = 0.3611 and *p* = 0.7694, respectively).

### Textbook Outcome Achievement

To comprehensively evaluate the quality of the surgical procedures, TO was analyzed as the primary composite endpoint for the matched cohort (*n* = 78). As summarized in Table 3, the overall TO achievement rate was 79.49% in the cRDG group and 87.18% in the rpRDG group, showing no statistically significant difference between the two approaches (*p* = 0.3621).

**Table 3:** Textbook outcomes after propensity score matching of patients who underwent robotic distal gastrectomy for gastric cancer: conventional versus reduced-port approach

Textbook outcomes	Conventional RDG (*n* = 39)	Reduced-port RDG (*n* = 39)	*p*-value
R0 resection	39 (100.00%)	39 (100.00%)	> 0.9999
LN dissection (≥ 16)	35 (89.74%)	38 (97.44%)	0.1655
No severe complications (≥ CD 2)	35 (89.74%)	35 (89.74%)	> 0.9999
No intervention	38 (97.44%)	37 (94.87%)	0.5560
No ICU readmission	39 (100.0%)	39 (100.0%)	> 0.9999
Hospital stay ≤ 21 days	37 (94.87%)	39 (100.0%)	0.1519
No readmission (≤ 30 days)	39 (100.00%)	38 (97.44%)	0.3142
No mortality (≤ 30 days)	39 (100.00%)	39 (100.00%)	> 0.9999
Overall TO achievement	31 (79.49%)	34 (87.18%)	0.3621

Variables are expressed as a number (%)

RDG = robotic distal gastrectomy; LN = lymph node; CD = Clavien-Dindo Classification; TO = Textbook outcome

Detailed analysis of the individual TO components revealed high performance in both groups. R0 resection and no ICU readmission were achieved in 100% of patients in both the cRDG and rpRDG groups (*p* > 0.9999). Regarding postoperative recovery and safety, there were no significant differences in the rates of no major complications (89.74% in both, *p* > 0.9999), no interventions (97.44% vs. 94.87%, *p* = 0.5560), and no 30-day mortality (100% in both, *p* > 0.9999). Furthermore, rates of hospital stay within 21 days (94.87% vs. 100.0%, *p* = 0.1519) and 30-day readmission-free discharge (100.0% vs. 97.44%, *p* = 0.3142) were comparable between groups.

### Postoperative analgesic consumption and pain analysis

The consumption of rescue analgesics was evaluated to assess postoperative analgesic requirements (Online Resource 3). No statistically significant differences were observed between the two groups regarding rescue analgesics administration. Repeated propacetamol hydrochloride administration (≥ 2 doses) was required in 7.69% of patients in the cRDG group and in none of the patients in the rpRDG group (*p* = 0.2403). Similarly, repeated pethidine hydrochloride administration (≥ 2 doses) was observed in 10.26% of the cRDG group and 5.13% of the rpRDG group (*p* = 0.6748).

Longitudinal changes in postoperative pain were analyzed using the NRS from POD 1 to POD 3 (Table 4). Post-hoc analyses identified significant differences at specific postoperative time points. On POD 1, pain scores were significantly lower in the rpRDG group than in the cRDG group (3.13 ± 0.15 vs. 2.62 ± 0.15, *p* = 0.0155). On POD 2, pain scores were comparable between the groups, with no statistically significant difference observed (2.72 ± 0.15 vs. 2.92 ± 0.15, *p* = 0.3260). By POD 3, both groups demonstrated similar reductions in pain scores (2.64 ± 0.12 vs. 2.38 ± 0.12, *p* = 0.1418). These findings suggest that the reduced-port approach may provide improved pain control during the immediate postoperative period, particularly on POD 1.

**Table 4:** Linear mixed-effects model for postoperative pain numeric rating scale scores: conventional versus reduced-port approach

**Panel A. Post-hoc comparisons of postoperative pain**			
**Time**	**cRDG (n = 39)**	**rpRDG (n = 39)**	**P-value**
POD 1	3.13 ± 0.15	2.62 ± 0.15	0.0155
POD 2	2.72 ± 0.15	2.92 ± 0.15	0.3260
POD 3	2.64 ± 0.12	2.38 ± 0.12	0.1418
**Panel B. Analysis of group, time, and group-by-time interaction effects on postoperative pain**			
**Effect**	**DF**	**F-statistic**	**P-value**
Group (cRDG vs. rpRDG)	1	1.66	0.2018
Time (POD 1 to 3)	2	4.98	0.0093
Group × Time interaction	2	5.83	0.0044

Variables are expressed as mean ± standard error

cRDG = conventional robotic distal gastrectomy; rpRDG = reduced-port robotic distal gastrectomy; POD = postoperative day

** P-values in Panel A were calculated using a linear mixed-effects model accounting for matched pairs

To evaluate postoperative pain trajectories over time, the linear mixed-effects model results are summarized in Table 4. A significant time effect was observed (*p* = 0.0093), indicating that pain intensity decreased over time in both groups. Although the overall group effect did not reach statistical significance (*p* = 0.2018), a significant group-by-time interaction was identified (*p* = 0.0044), suggesting that the pattern of pain reduction over time differed significantly between the cRDG and rpRDG groups.

## Discussion

This study demonstrates that rpRDG is technically feasible and oncologically safe, with a statistically significant reduction in immediate postoperative pain on POD 1 observed as a secondary finding. Using a rigorous methodology involving CUSUM analysis to mitigate learning curve bias and PSM to ensure cohort comparability, we found that both approaches achieved equivalent surgical quality, as evidenced by comparable TO achievement rates (87.18% for rpRDG vs. 79.49% for cRDG, *p* = 0.3621). Additionally, the linear mixed-effects model revealed a significant interaction between surgical group and time (*p* = 0.0044), indicating that the trajectory of pain recovery differed significantly between groups, with the rpRDG group demonstrating lower pain intensity in the immediate postoperative period, suggesting a pain-reducing effect of the reduced-port approach. These findings suggest that the reduced-port configuration may serve as a valuable clinical strategy in the transition toward less invasive robotic surgery for GC.

A major concern with reduced-port surgery is whether limited instrument manipulation compromises oncologic radicality [4, 10, 18, 19]. In this study, lymphadenectomy quality—a cornerstone of curative gastrectomy—was preserved in the rpRDG group. After covariate adjustment, no significant differences emerged between rpRDG and cRDG in D2 lymphadenectomy completion rates, retrieved lymph node counts, or R0 resection rates. These observations suggest that rpRDG achieves oncologic adequacy equivalent to cRDG. This technical success likely stems from the robotic system’s multi-articulated instruments, which compensate for reduced triangulation in limited-port settings and enable precise dissection around major vessels without increasing blood loss [20, 21].

Contrary to expectations that port reduction would prolong operative time due to instrument collision, operative time in the rpRDG was comparable to that in the cRDG. This finding can be interpreted in the context of both the ergonomic advantages of the robotic platform and the surgeon’s accumulated experience. Robotic assistance decouples port placement from surgeon posture, maintaining stable control and procedural efficiency even with fewer ports [4, 22]. The CUSUM analysis provided critical insights into the technical feasibility of rpRDG. In this study, the turning point for the rpRDG group was reached at the 15th case, which was significantly earlier than the 30th case observed for the cRDG group (Online resource 1). This accelerated attainment of proficiency in the rpRDG group is likely attributable to a “proficiency transfer” from the surgeon’s established expertise in conventional robotic gastrectomy. Given this proficiency transfer, the exclusion of early rpRDG cases was deemed unnecessary, as the technical foundation established through cRDG experience was considered sufficient to ensure procedural stability from the outset of rpRDG adoption. The substantially lower maximum CUSUM value in the rpRDG group (364.30) compared to the cRDG group (1168.67) further suggests a reduced initial learning burden when transitioning to the reduced-port configuration. Methodologically, identifying these thresholds was essential for mitigating procedural bias. Since our earliest robotic experiences were predominantly concentrated in the cRDG group, there was a high risk that the learning curve effect could have negatively skewed the outcomes of the conventional approach or masked the true benefits of the newer rpRDG technique. By excluding the initial 30 cRDG cases based on the CUSUM inflection point, we ensured that the subsequent comparative analysis focused exclusively on the stable proficiency phase. The significant reduction in operative times observed in both groups after their respective turning points (*p* < 0.001 for cRDG and *p* = 0.0215 for rpRDG) underscores that once a surgeon is past the initial learning phase, the reduced-port approach can be performed with efficiency and safety comparable to the conventional method. Collectively, these findings demonstrate that rpRDG imposes no additional technical burden for an experienced robotic surgeon and that mastery of conventional robotic gastrectomy mitigates challenges associated with port reduction [20].

Regarding postoperative pain control, the consumption of rescue analgesics was comparable between the two groups. Repeated administration of propacetamol (≥ 2 doses) was less frequent in the rpRDG group (7.69% vs. 0%), although this difference did not reach statistical significance (*p* = 0.2403). However, a linear mixed-effects model revealed a significant interaction between the surgical approach and time (*p* = 0.0044), indicating distinct pain recovery patterns. Specifically, the rpRDG group reported significantly lower pain NRS scores on POD 1 compared to the cRDG group (3.13 ± 0.15 vs. 2.62 ± 0.15, *p* = 0.0155). While the cRDG group showed a gradual decline from an initially higher pain level, the rpRDG group maintained lower early-phase pain, although a transient, non-significant increase was noted on POD 2. The eventual convergence of pain scores and the lack of overall difference in rescue analgesic use may be attributed to the “dominant incision effect.” [8, 10]. The 2–3 cm umbilical mini-laparotomy—the primary source of somatic pain for specimen extraction—remained identical in both approaches, potentially masking the full benefit of eliminating ancillary trocars in the later postoperative phase. Nevertheless, the significant reduction in POD 1 pain suggests a measurable early recovery benefit of the reduced-port approach, particularly within the context of an already-optimized ERAS protocol where large intergroup differences are inherently constrained. Notably, Park et al. reported that the da Vinci SP system can be utilized via a Pfannenstiel incision for both port placement and specimen extraction, potentially eliminating the umbilical incision entirely and offering advantages in cosmesis, postoperative pain reduction, and incisional hernia prevention [20]. While our study employed an umbilical approach even in SP cases, such alternative extraction strategies may represent a meaningful direction for further reducing the dominant incision effect in future iterations of reduced-port robotic gastrectomy. The observed pain benefit should not be interpreted in isolation as a justification for SP system adoption; rather, it represents one incremental finding within a broader feasibility framework, with definitive cost-benefit conclusions requiring prospective, multicenter evaluation.

The stepwise reduction from five to two ports was achieved through iterative refinement of surgical techniques. The marionette technique using endoclips was initially implemented to compensate for assistant port loss in four-port cRDG and was later incorporated as a core element of rpRDG, enabling stable static retraction while reserving robotic arms for active dissection [23]. During robotic gastrectomy with the da Vinci SP system, several technical adjustments were introduced to accommodate the unique characteristics of the platform and facilitate stable surgical performance. A “single-plus-one” approach permitted the use of laparoscopic staplers and advanced energy devices in the absence of SP-dedicated instruments [12], whereas refined bipolar dissection techniques—including the double-bipolar method with soft coagulation—were adapted from established multiport robotic protocols to ensure precise and controlled tissue handling [24, 25]. Additionally, flexible scope adjustment, particularly switching to an “above-view” during suprapancreatic dissection, countered the inherent below-view limitation of the SP system and allowed anatomical recognition comparable to that of conventional multiport surgery [4, 11]. A supplementary video clip illustrating suprapancreatic lymph node dissection—the core operative component of rpRDG—is provided to enhance procedural understanding (**Online Resource 4**).

The clinical rationale for rpRDG extends beyond perioperative outcome comparisons. First, this approach maximizes the use of the unavoidable umbilical incision for specimen extraction by repurposing it as the primary access port, thereby obviating the need for supplementary lateral abdominal wall incisions dedicated solely to access [18, 26, 27]. Second, although the present study lacked power to detect differences in rare wound-related complications, fewer ports may theoretically decrease the risk of surgical site infection, port-site hernia, and hematoma by reducing parietal entry sites [6]. Finally, rpRDG aligns with ongoing efforts to prioritize patient-centered surgical innovation. By preserving oncologic adequacy while minimizing parietal trauma and visible scarring, this technique may improve patient experience and cosmetic outcomes without jeopardizing oncologic safety [9, 22, 28–31].

Critics may argue that the clinical benefits of rpRDG observed in this study—specifically the reduction in POD 1 pain without a significant decrease in rescue analgesic frequency—could be perceived as modest. However, these findings must be interpreted within the context of the current surgical landscape. LG has already achieved a high plateau of clinical excellence, characterized by remarkably low complication rates and standardized outcomes. While previous studies have suggested that robotic platforms can further reduce complications, the baseline incidence is already so low that achieving a statistically superior “major” breakthrough is increasingly challenging. Given the substantially higher costs associated with robotic systems compared to conventional laparoscopy, it is imperative to leverage the platform’s technical precision to secure “marginal gains” that are otherwise difficult to attain. In this regard, the significant reduction in immediate postoperative pain (POD 1) represents a meaningful incremental benefit. Although the observed 0.51-point NRS difference on POD 1 falls below the commonly cited minimal clinically important difference (MCID) threshold of 1–2 points for acute pain, interpreting this finding solely through a single-timepoint MCID lens may not fully capture the nature of the result. The primary pain finding is not the magnitude of the POD 1 difference in isolation, but rather the significant group-by-time interaction (*p* = 0.0044), which demonstrates that the two groups followed fundamentally different pain recovery trajectories—a dimension not directly addressed by single-timepoint MCID thresholds. By refining the surgical approach through the reduced-port technique, we can optimize the value proposition of the robotic system, moving beyond mere safety to enhance the quality of early recovery and patient-reported outcomes.

Several considerations should be taken into account when interpreting the results. This analysis was retrospective and based on procedures performed by a single surgeon at a single center, which may restrict the external applicability of the findings despite the use of relatively consistent surgical techniques. Another primary limitation of this study is the technological heterogeneity within the rpRDG cohort, which comprised both multi-port-based platforms (da Vinci Xi and da Vinci 5) and a single-port platform (da Vinci SP). Because these systems dictate different intraoperative kinematics, articulation angles, and assistant port utilization, merging them into a single cohort may introduce confounding factors. Although our exploratory subgroup analysis demonstrated comparable short-term surgical and oncological outcomes between the Xi/V and SP subgroups (**Online Resource 5**), the statistical power was inherently insufficient to draw definitive conclusions given the small SP sample size (*n* = 7). Future well-designed, large-scale prospective studies strictly separating multi-port and single-port configurations are warranted to comprehensively evaluate platform-specific outcomes in reduced-port robotic gastrectomy. Additionally, DM and Dyslipidemia showed baseline imbalances that could not be fully addressed by PSM, as their inclusion as matching covariates would have substantially reduced the number of achievable pairs. While the comparable TO outcomes observed despite a higher comorbidity burden in the rpRDG group provide some reassurance against favorable selection bias, this imbalance remains an unmeasured confounder and should be interpreted accordingly. Furthermore, the numerically higher TO rate observed in the rpRDG group should be interpreted with caution. Although CUSUM analysis and PSM were applied to mitigate learning-curve and selection bias, two sources of residual confounding cannot be fully excluded: first, chronological bias arising from the concentration of rpRDG cases in the latter study period, during which institutional improvements in ERAS compliance and perioperative team coordination may have conferred an unmeasured benefit; and second, anatomical selection bias, as PSM adjustment for BMI cannot capture intra-abdominal difficulty variables such as visceral fat area, tissue friability, or adhesion severity. These factors preclude causal interpretation of the numerical TO difference, and the finding should be understood strictly in the context of statistical equivalence (*p* = 0.3621). Finally, the post-matching sample size of 78 patients is likely underpowered to detect rare port-related complications such as incisional hernias or wound-site infections. As the study was designed and powered for TO equivalence as the primary endpoint, the absence of observed differences in these rare events should not be interpreted as evidence of equivalence in this regard. Definitive assessment of port-site specific morbidity will require large-scale, multicenter studies with extended follow-up. Thus, this study does not propose rpRDG as a new standard but rather establishes its technical feasibility and oncologic adequacy during the transition from multiport to reduced-port robotic gastrectomy. By demonstrating meaningful marginal gains in early recovery despite the high cost of the robotic platform, this work provides a practical foundation for future large-scale, multicenter trials.

## Conclusion

rpRDG demonstrated comparable surgical and oncologic safety to cRDG, achieving equivalent TO rates as the primary endpoint. As a secondary finding, a statistically significant reduction in POD 1 pain was observed, representing a meaningful marginal gain in an era of highly standardized surgical outcomes. These results suggest that rpRDG is a promising, patient-centered alternative for early recovery, warranting further validation through large-scale multicenter studies.

## Supplementary Information

Below is the link to the electronic supplementary material.

Supplementary Material 1

Supplementary Material 2

Supplementary Material 3

Supplementary Material 4

Supplementary Material 5

## Data Availability

No datasets were generated or analysed during the current study.
