# Supplementary material for: Comparison of textbook outcomes and postoperative pain trajectories between reduced-port and conventional robotic distal gastrectomy: a cumulative sum (CUSUM)-adjusted propensity score-matched analysis
Source: J Robot Surg. 2026 Jun 16;20(1):588. doi: 10.1007/s11701-026-03607-y (PMC13269504; doi:10.1007/s11701-026-03607-y)
Supplement: Supplementary file 1 — Supplementary Material 1 [file 11701_2026_3607_MOESM1_ESM.docx]

**Journal of Robotic Surgery**

**Title**

Comparison of textbook outcomes and postoperative pain trajectories between reduced-port and conventional robotic distal gastrectomy: A cumulative sum (CUSUM)-adjusted propensity score-matched analysis

**Authors**

Dongwon Lim, MD, PhD^1,2,3^; Jongmin Han, MD^2^; Juyrong Noh, MD^2^; Si-Hak Lee, MD, PhD^1,2,3^; Sun-Hwi Hwang, MD, PhD^1,2,3^; Hanpyo Hong, PhD^3^; Jae Hun Chung, MD, PhD^1,2,3*^

**Affiliations**

^1^ Department of Surgery, Pusan National University Yangsan Hospital, Republic of Korea

^2^ School of Medicine, Pusan National University, Yangsan, Republic of Korea

^3^ Research Institute for Convergence of Biomedical Science and Technology, Pusan National University Yangsan Hospital, Yangsan, Republic of Korea

**Corresponding author**

Jae Hun Chung, MD, PhD

Department of Surgery, Pusan National University Yangsan Hospital, 20 Geumo-ro, Mulgeum-eup, Yangsan-si, Kyungsangnam-do 50612, Republic of Korea

TEL: +82-55-360-2124

E-mail: jhchung@pnuyh.co.kr

**Online Resource 3.** Postoperative rescue analgesic consumption: conventional versus reduced-port approach

| **Frequency of additional analgesic administration** | **Conventional**  **RDG**  **(n=39)** | **Reduced-port**  **RDG**  **(n=39)** | ***p*-value** |
| --- | --- | --- | --- |
| Propacetamol hydrochloride |  |  | 0.2403 |
| < 2 | 36 (92.31 %) | 39 (100.00 %) |  |
| ≥ 2 | 3 (7.69 %) | 0 (0.00 %) |  |
| Pethidine hydrochloride |  |  | 0.6748 |
| < 2 | 35 (89.74 %) | 37 (94.87 %) |  |
| ≥ 2 | 4 (10.26 %) | 2 (5.13 %) |  |

Variables are expressed as a number (%)

RDG = robotic distal gastrectomy
