# Supplementary figures and images for: Comparison of textbook outcomes and postoperative pain trajectories between reduced-port and conventional robotic distal gastrectomy: a cumulative sum (CUSUM)-adjusted propensity score-matched analysis
Source: J Robot Surg. 2026 Jun 16;20(1):588. doi: 10.1007/s11701-026-03607-y (PMC13269504; doi:10.1007/s11701-026-03607-y)

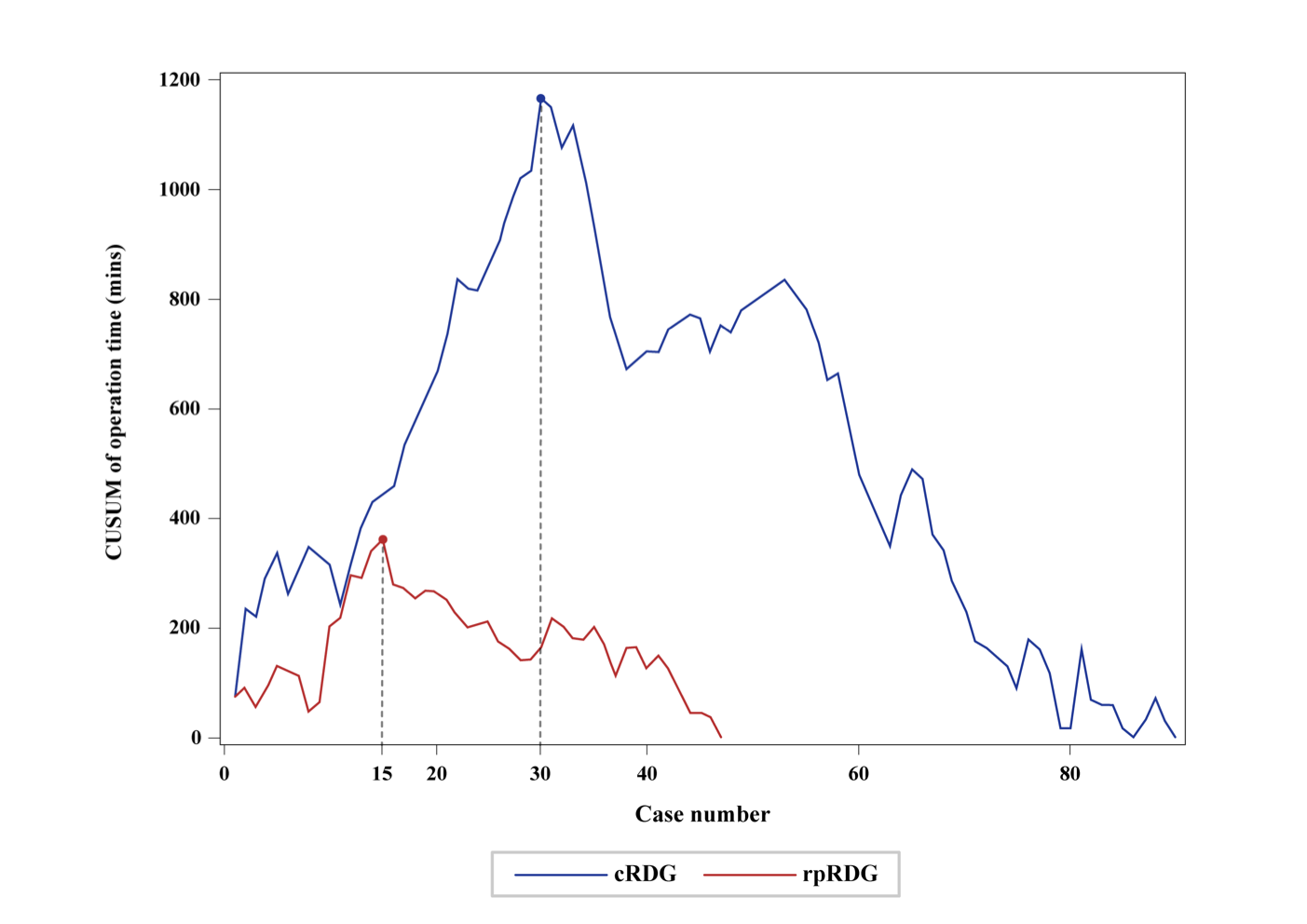

Supplement: Supplementary file 2 — Supplementary Material 2 [file 11701_2026_3607_MOESM2_ESM.tiff]

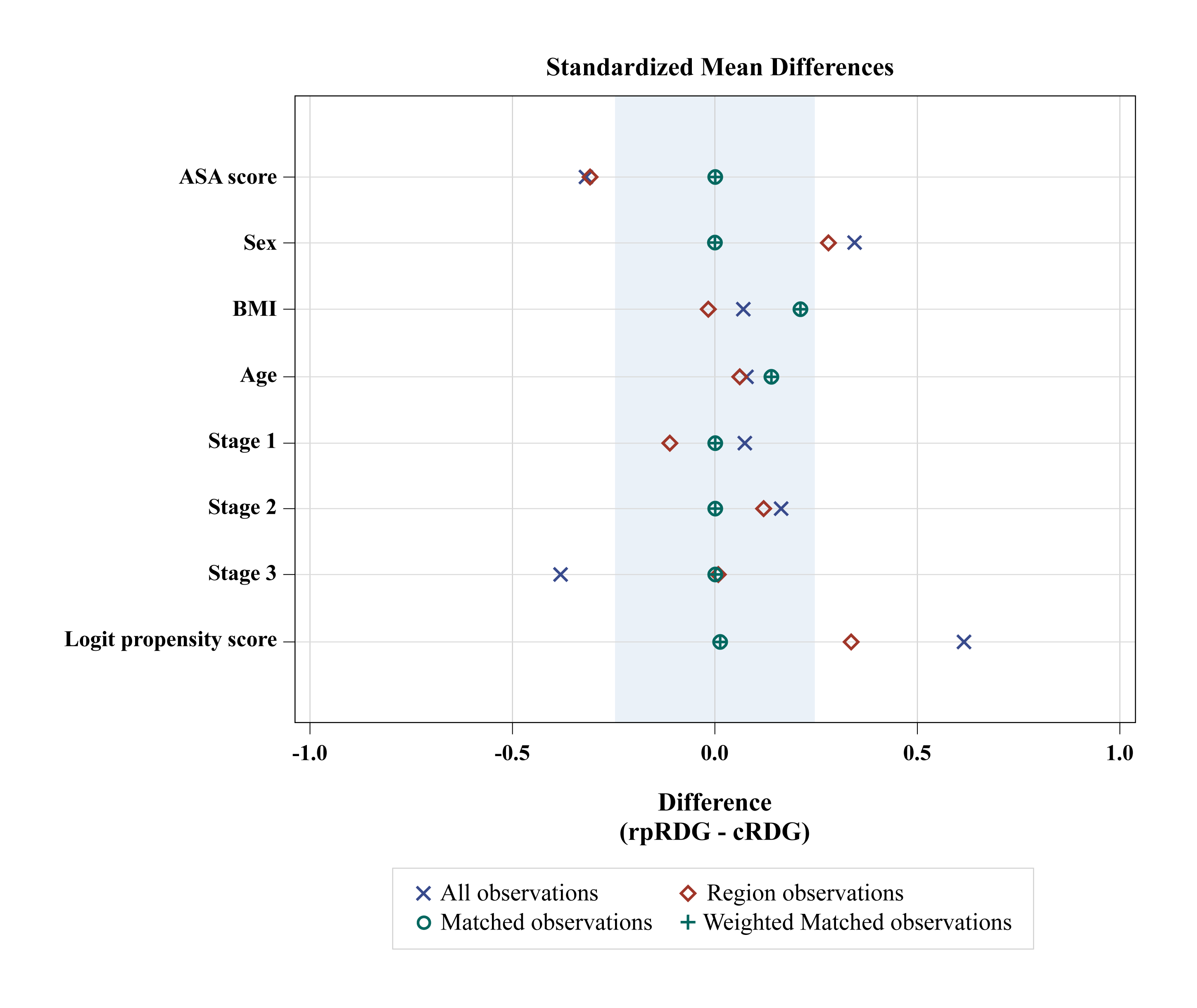

Supplement: Supplementary file 3 — Supplementary Material 3 [file 11701_2026_3607_MOESM3_ESM.tiff]
