# Supplementary material for: Comparison of textbook outcomes and postoperative pain trajectories between reduced-port and conventional robotic distal gastrectomy: a cumulative sum (CUSUM)-adjusted propensity score-matched analysis
Source: J Robot Surg. 2026 Jun 16;20(1):588. doi: 10.1007/s11701-026-03607-y (PMC13269504; doi:10.1007/s11701-026-03607-y)
Supplement: Supplementary file 5 — Supplementary Material 5 [file 11701_2026_3607_MOESM5_ESM.docx]

# Online Resource 5. Subgroup analysis within the reduced-port robotic distal gastrectomy cohort: Xi/V vs. SP

| **Variables** | **Xi/V Subgroup**  **(n=32)** | **SP Subgroup**  **(n=7)** | ***p*-value** |
| --- | --- | --- | --- |
| Age (years) | 63.3 ± 9.0 | 69.4 ± 4.5 | 0.0607 |
| BMI (kg/m²) | 24.7 ± 2.9 | 23.0 ± 2.4 | 0.1506 |
| Sex |  |  | 1.0000 |
| Male | 20 (62.5%) | 5 (71.4%) |  |
| Female | 12 (37.5%) | 2 (28.6%) |  |
| ASA score |  |  | 0.8872 |
| < 3 | 27 (84.4%) | 6 (85.7%) |  |
| ≥ 3 | 5 (15.6%) | 1 (14.3%) |  |
| Operation time (min) | 245.1 ± 35.3 | 251.6 ± 36.7 | 0.3794 |
| Estimated blood loss (mL) | 46.4 ± 38.3 | 28.6 ± 10.7 | 0.2570 |
| Retrieved LN count | 40.4 ± 17.5 | 32.3 ± 15.5 | 0.2201 |
| Textbook Outcome (TO) |  |  | 1.0000 |
| Achieved (Yes) | 28 (87.5%) | 6 (85.7%) |  |
| Not achieved (No) | 4 (12.5%) | 1 (14.3%) |  |
